# A CHD8-TRRAP axis facilitates MYC and E2F target gene regulation in human neural stem cells

**DOI:** 10.1016/j.isci.2025.111978

**Published:** 2025-02-12

**Authors:** Lize Meert, Mariana Pelicano de Almeida, Mike R. Dekker, Dick H.W. Dekkers, Karol Nowosad, Danny Huylebroeck, Mirjam van den Hout, Zeliha Ozgür, Wilfred F.J. van IJcken, Jeroen Demmers, Maarten Fornerod, Raymond A. Poot

**Affiliations:** 1 Department of Cell Biology, Erasmus MC, Wytemaweg 80, 3015 CN Rotterdam, the Netherlands; 2 Center for Proteomics, Erasmus MC, 3015 CN Rotterdam, the Netherlands; 3 Center for Biomics, Erasmus MC, 3015 CN Rotterdam, the Netherlands

**Keywords:** Molecular biology, Molecular Genetics, Stem cells research

## Abstract

Mutations in ATP-dependent chromatin remodeler CHD8 cause one of the most frequent monogenetic forms of autism and are associated with brain overgrowth. Nevertheless, the activities of CHD8 in autism-relevant cell types are still poorly understood. Here, we purify the CHD8 protein from human neural stem cells and determine its interaction partners using mass spectrometry. We identify the TRRAP complex, a coactivator of MYC and E2F transcription factors, as a prominent CHD8 interaction partner. CHD8 colocalizes genome-wide with TRRAP and binds together at MYC and E2F target gene promoters in human neural stem cells. Depletion of CHD8 or TRRAP in human neural stem cells shows downregulation of MYC and E2F target genes as the most prominent gene-regulatory events. Depletion of CHD8 diminishes cell-cycle entry into S-phase. MYC and E2F transcription factors are established oncogenes and regulate cell growth. Our results link CHD8 to TRRAP in facilitating the regulation of MYC and E2F target genes in human neural stem cells.

## Highlights

• CHD8 and TRRAP interact
• CHD8 and TRRAP colocalize on the genome
• CHD8 and TRRAP regulate MYC and E2F target genes
• CHD8 knockdown causes diminished entry into S-phase

## Graphical abstract

**Figure:**
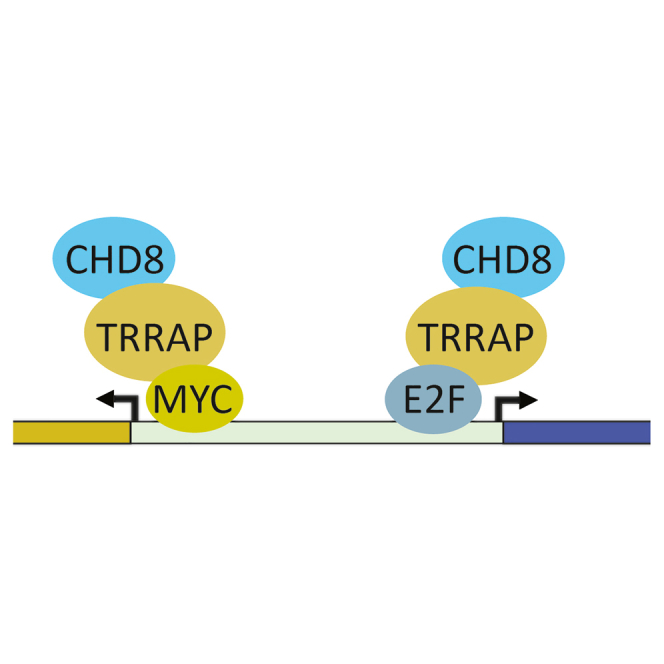

## Introduction

Autism spectrum disorders (ASDs) are a heterogeneous group of neurodevelopmental disorders, characterized by deficits in social communication skills and the occurrence of repetitive sensory-motor behavior, and this often coincides with impaired cognitive functions.^1^ Development of ASD has a high genetic etiology.^2^^,^^3^ Chromodomain helicase DNA-binding protein 8 (*CHD8*) is one of the first genes where mutations were identified as a monogenic cause of autism^4^^,^^5^^,^^6^ and to date *CHD8* is in the top 5 of the most frequently mutated genes in autism patients.^2^^,^^7^^,^^8^^,^^9^ Autism patients with *CHD8* mutations have a distinct clinical phenotype, which often includes brain overgrowth.^8^^,^^10^ A brain overgrowth phenotype is also observed in *Chd8* heterozygous knockout and *Chd8* hypomorph mouse models.^11^^,^^12^^,^^13^ The effect of *Chd8* mutations on mouse brain development appears to be dose-dependent, as more pronounced depletion of CHD8 negatively affects the neural stem cell (NSC) proliferation and causes mouse brain hypoplasia.^13^^,^^14^ Moreover, *Chd8* heterozygous knockouts in mice display small, but widespread, changes in gene expression.^11^^,^^12^ In human brain organoids harboring heterozygous *CHD8* loss-of-function mutations, there is a marked overgrowth phenotype and a disrupted balance of the generation of excitatory and inhibitory neurons.^15^^,^^16^ *CHD8* haploinsufficiency in human neural stem cells (hNSCs) shortens the G1 phase and increases the proliferation speed.^17^ Depletion of CHD8 in both mouse and human NSCs causes dysregulation of other autism genes.^18^^,^^19^^,^^20^

CHD8 is a chromatin remodeler that uses ATP to mobilize nucleosomes and is a member of the family of the chromodomain-helicase-DNA binding (CHD) proteins that consists of nine members (CHD1-9).^21^ Since CHD proteins have no sequence specificity, they rely on chromatin features or other protein factors for their specific targeting to the genome, which in case of the CHD8 protein leads to multiple suggested functions.^21^ CHD8 was shown to directly bind β-catenin and suppress Wnt signaling by inhibiting the binding of β-catenin to TCF.^22^^,^^23^ In addition, it has also been demonstrated that CHD8 facilitates RNA polymerase II- and III-mediated transcription^24^^,^^25^ and regulates the transcription of G1/S transition genes.^26^ Furthermore, CHD8 is implicated in the suppression of p53-dependent apoptosis^27^^,^^28^ and aids BRD4 in leukemia maintenance.^29^

Here, we identified the CHD8 interactome in hNSCs and found the TRRAP complex as a novel and prominent interactor of CHD8. The TRRAP complex is the main co-activator of MYC and E2F transcription factors^30^^,^^31^^,^^32^ and is essential for their role in the stimulation of cell proliferation and oncogenic transformation.^30^^,^^33^ TRRAP is important for proliferation of mouse NSCs.^32^ Missense mutations in *TRRAP* also cause an intellectual disability syndrome with various clinical features, including autism.^34^ We find here that the most prominent gene regulatory activity of CHD8 and TRRAP in hNSCs is to together bind and activate MYC and E2F target genes.

## Results

### The protein interactome of CHD8 in human neural stem cells features the TRRAP complex

**Figure 1:**
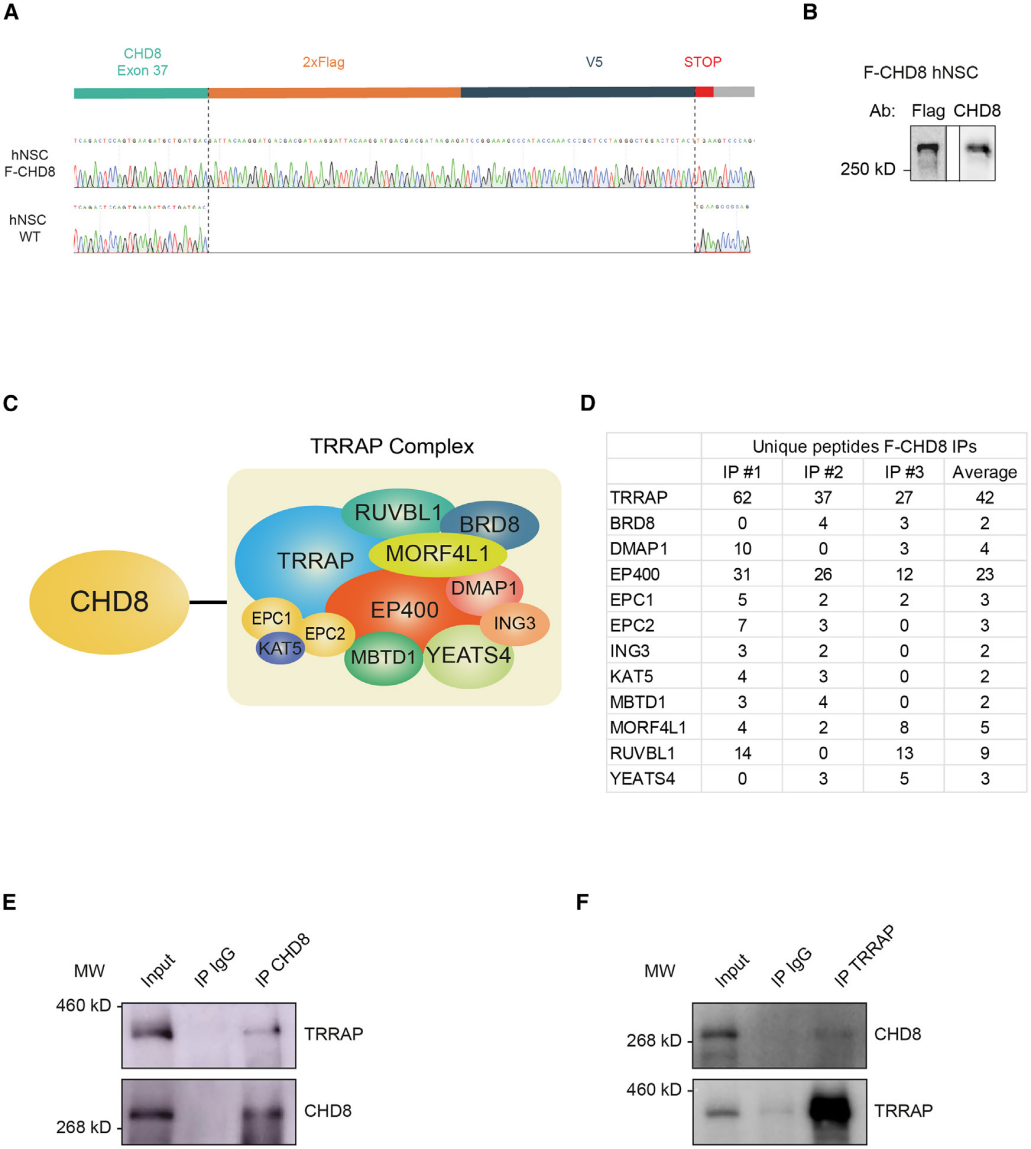
CHD8 interactome identifies TRRAP complex as a CHD8 interaction partner in hNSCs (A) Schematic view of the insertion of 2xFlag and V5 tag at the C-terminus of both alleles of *CHD8*. (B) Western blot analyses with anti-Flag antibody (left) and CHD8 antibody (right) showing expression of F-CHD8. (C) Schematic view of TRRAP complex subunits observed in multiple purifications of F-CHD8. (D) Number and average of unique peptides of TRRAP subunits observed by mass spectrometry analyses of 3 immunoprecipitations (IP) of F-CHD8. (E) Western blot analyses of immunoprecipitations with CHD8 antibody or an IgG control. TRRAP (upper panel) and CHD8 (lower panel) are detected by TRRAP antibody and CHD8 antibody, respectively. Molecular weight (MW) markers are indicated. (F) Western blot analyses of immunoprecipitations with TRRAP antibody or an IgG control. CHD8 (upper panel) and TRRAP (lower panel) are detected by CHD8 antibody and TRRAP antibody, respectively. Molecular weight (MW) markers are indicated.

Interaction partners can provide insight into the cellular function of proteins. To identify the interaction partners of endogenously expressed CHD8 in hNSCs, we inserted DNA for a double FLAG-affinity tag into the C-terminal end of the genomic open reading frame of *CHD8* using the CRISPR-Cas 9 system (Figure 1A). A clone that contained a double FLAG tag and a V5-tag in both *CHD8* alleles, F-CHD8 hNSCs, was identified by DNA sequencing and anti-FLAG western blot (Figures 1A and 1B). V5 chromatin immunoprecipitation (ChIP) in F-CHD8 hNSCs showed that the tagged CHD8 protein was still functional and could bind to known CHD8 target promoters (Figure S1A). Three independent batches of nuclear extract, each from several hundred million hNSCs, were obtained from both F-CHD8 hNSCs and control hNSCs. Subsequently, three independent anti-FLAG affinity purifications from F-CHD8 hNSCs and control hNSCs were performed, as previously described.^35^^,^^36^ Using mass spectrometry, we identified 24 CHD8 interactors that were present in all three F-CHD8 purifications and another 96 that were present in two out of three purifications. All identified interaction partners were at least 3-fold enriched compared to control purifications (Table S1, STAR Methods). We detected CHD7 and ZNF143, previously known interactors of CHD8.^24^^,^^37^ Prominent and novel among the CHD8 interactors was the TRRAP complex^38^ with subunits TRRAP, EP400, EPC1 and MORF4L detected in all 3 purifications and subunits BRD8, DMAP1, KAT5, EPC2, ING3, MBTD1, RUVBL1, and YEATS4 identified in 2 purifications (Figures 1C and 1D and Table S1). In the MaxQuant analysis of the mass spectrometry data TRRAP, EP400, YEATS4, MORF4L1, BRD8, DMAP1 and RUVBL2 make the FDR<0.05 cut-off (Table S2). To verify the CHD8-TRRAP interaction, we performed immunoprecipitations (IPs) of endogenous TRRAP and endogenous CHD8 in hNSC nuclear extracts, using a TRRAP antibody and a CHD8 antibody, respectively. CHD8 IP precipitated CHD8 and co-precipitated TRRAP (Figure 1E). Conversely, TRRAP IP precipitated TRRAP and co-precipitated CHD8 (Figure 1F). We conclude that CHD8 and the TRRAP complex are *bona fide* interaction partners in hNSCs.

### CHD8 colocalizes with TRRAP on the human neural stem cell genome

**Figure 2:**
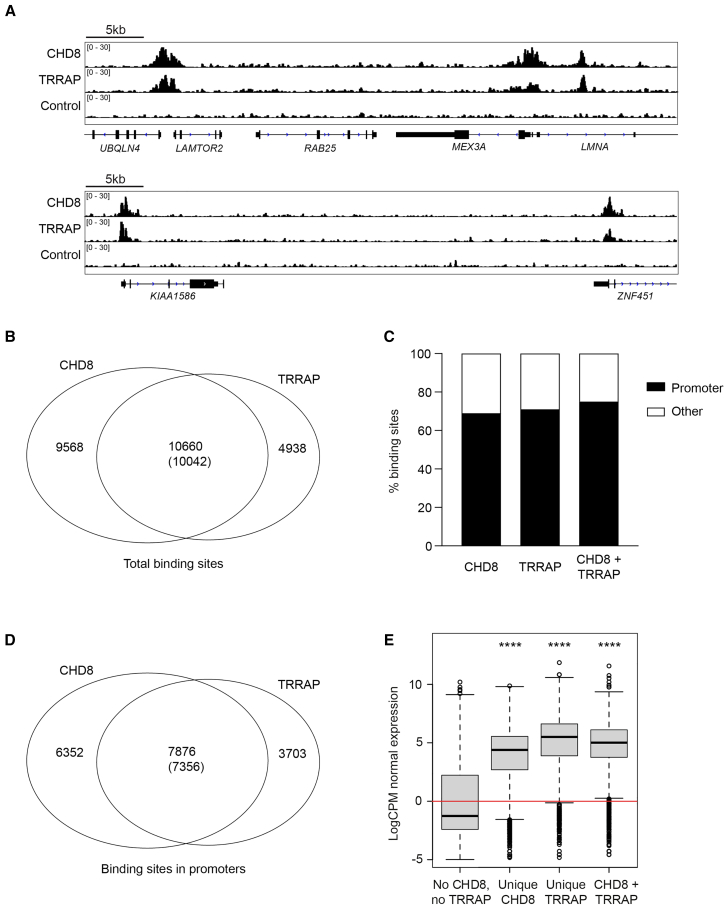
CHD8 and TRRAP highly overlap in genome-wide binding sites (A) CHD8 and TRRAP often colocalize on the hNSC genome. Representative ChIP-seq tracks for CHD8 and TRRAP and an IgG control ChIP-seq. Range of reads per million per base pair is indicated on the Y axis. Genes are indicated. Scale bar depicts 5kb. (B) Venn diagram of CHD8 and TRRAP significant genome binding sites, as determined by ChIP-seq. Numbers of TRRAP and CHD8 binding sites, overlapping or not, are indicated. The number of overlapping TRRAP binding sites (without brackets) is different from the number of overlapping CHD8 binding sites as multiple sites of TRRAP can overlap with one site of CHD8 and vice versa. Overlap *p* = 0 by hypergeometric test with the number of possible binding places equal the sum all detected peaks. (C) CHD8 and TRRAP predominantly bind to promoters. Bar diagrams of the percentages of CHD8 genome binding sites (left), TRRAP binding sites (middle) and CHD8-TRRAP overlapping binding sites (right) in promoters (black) or outside (white) promoters (+/−1 kb from transcription start site). (D) Venn diagram of CHD8 and TRRAP significant binding sites at promoters. Numbers of TRRAP and CHD8 binding sites, overlapping or not, are indicated. The number of overlapping TRRAP binding sites (without brackets) is different from the number of overlapping CHD8 binding sites as multiple sites of TRRAP can overlap with one site of CHD8 and vice versa. Overlap *p* = 0 by hypergeometric test with the number of possible binding places equal the sum all detected peaks. (E) Expression levels of genes bound by CHD8 and TRRAP at their promoters. Boxplots of log CPM of expression of genes with no CHD8 or TRRAP binding sites (No CHD8, no TRRAP), only CHD8 binding sites (Unique CHD8), only TRRAP binding sites (Unique TRRAP) or CHD8 and TRRAP overlapping binding sites at their promoter (CHD8 + TRRAP). Expression from promoters with no CHD8 and TRRAP present is significantly lower (Wilcoxon test) than from promoters with unique CHD8 (*p*-value = 1.8 e^−12^), promoters with unique TRRAP (*p*-value <2.2 e^−16^) or promoters with CHD8 and TRRAP (*p*-value <2.2 e^−16^). P-values <0.0001 are indicated with ∗∗∗∗.

Our biochemical interaction data showed that CHD8 and the TRRAP complex interact, which may suggest that they cooperate in their biological function. To test whether CHD8 cooperates with TRRAP in transcriptional regulation, we first determined the genome-wide binding sites of CHD8 and TRRAP in hNSCs by ChIP-seq with antibodies against CHD8 or TRRAP. ChIP-seq tracks for CHD8 and TRRAP showed specific enrichments, with a high degree of overlap between CHD8 and TRRAP binding sites (Figure 2A). We found 19610 significant binding sites for CHD8 and 15598 significant binding sites for TRRAP, which show an overlap of 10660 TRRAP binding sites, 68% of the total number of TRRAP binding sites (Figure 2B). Both CHD8 and TRRAP localized predominantly at promoters (Figure 2C). CHD8 and TRRAP binding sites at promoters overlapped at 68% of all TRRAP sites at promoters and at 54% of all CHD8 sites at promoters (Figure 2D). The promoter binding of CHD8 or TRRAP positively correlated with corresponding gene expression levels in hNSCs (Figure 2E). Our CHD8 binding sites overlap nearly completely with CHD8 binding sites in human neural progenitors previously determined^18^ (Figure S1B).

### CHD8 and TRRAP colocalize at promoters of MYC and E2F target genes

**Figure 3:**
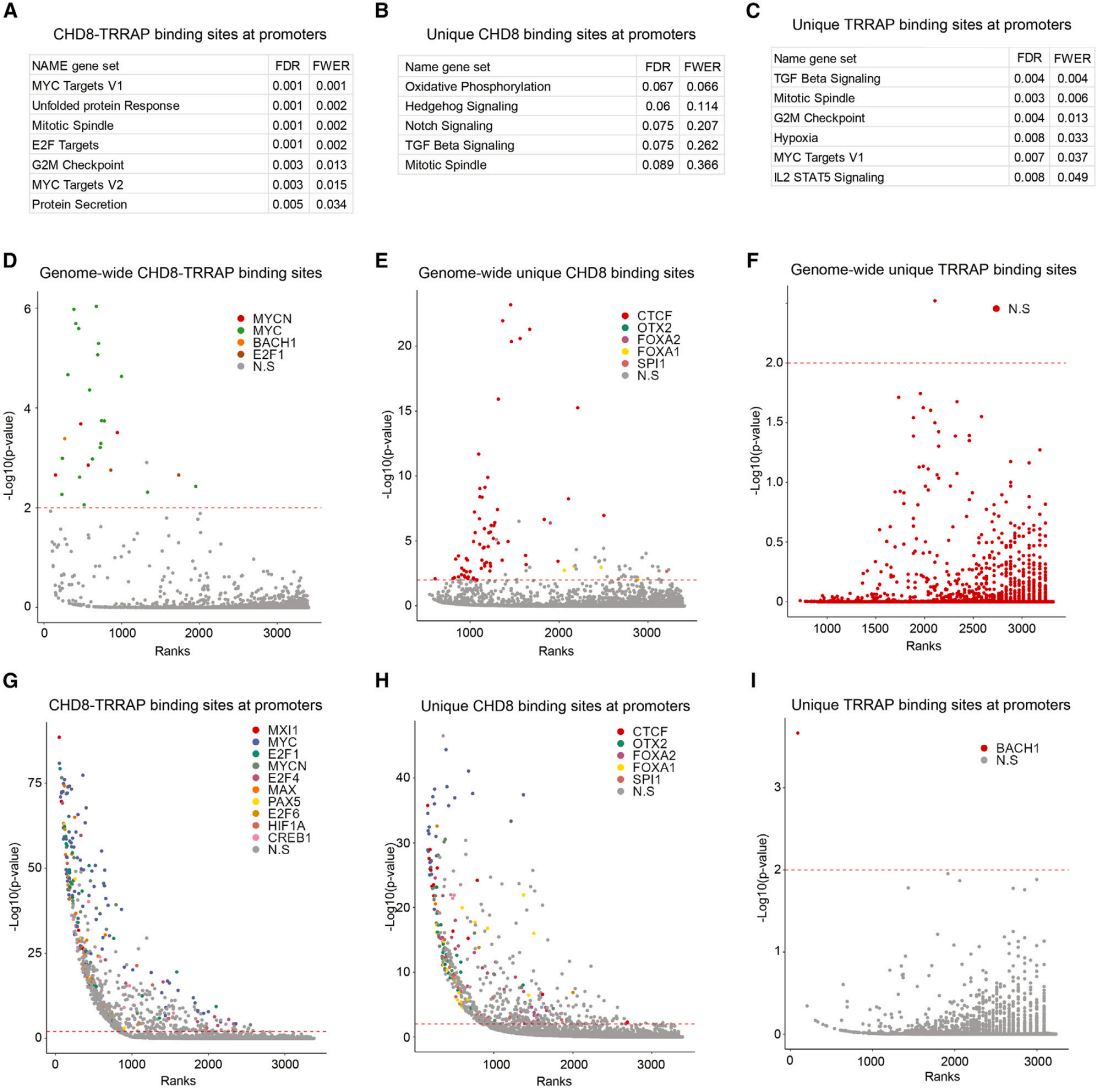
CHD8 and TRRAP bind to promoters of MYC and E2F target genes (A) Gene Set enrichments for promoters with overlapping CHD8 and TRRAP binding sites. False discovery rate q-value (FDR) and family-wise error rate *p*-value (FWER) are indicated. (B) Gene Set enrichments for promoters with unique CHD8 binding sites. False discovery rate q-value (FDR) and family-wise error rate *p*-value (FWER) are indicated. (C) Gene Set enrichments for promoters with unique TRRAP binding sites. False discovery rate q-value (FDR) and family-wise error rate *p*-value (FWER) are indicated. (D) Enrichment of transcription factor binding sites at overlapping genome-wide CHD8 and TRRAP binding sites. Transcription factor ChIP-seq experiments in the UniBind database are ranked on the X axis and ChIP-seq binding patterns that overlap are colored according to the indicated corresponding transcription factor. Overlap significance threshold and –log10(*p*-value) of the overlap are indicated on the Y axis. (E) Enrichment of transcription factor binding sites at unique genome-wide CHD8 binding sites. Transcription factor ChIP-seq experiments in the UniBind database are ranked on the X axis and ChIP-seq binding patterns that overlap are colored according to the indicated corresponding transcription factor. Overlap significance threshold and –log10(*p*-value) of the overlap are indicated on the Y axis. (F) Enrichment of transcription factor binding sites at unique genome-wide TRRAP binding sites. Transcription factor ChIP-seq experiments in the UniBind database are ranked on the X axis and ChIP-seq binding patterns that overlap are colored according to the indicated corresponding transcription factor. Overlap significance threshold and –log10(*p*-value) of the overlap are indicated on the Y axis. (G) Enrichment of transcription factor binding sites at overlapping CHD8 and TRRAP binding sites at promoters. Transcription factor ChIP-seq experiments in the UniBind database are ranked on the X axis and ChIP-seq binding patterns that overlap are colored according to the indicated corresponding transcription factor. Overlap significance threshold and –log10(*p*-value) of the overlap are indicated on the Y axis. (H) Enrichment of transcription factor binding sites at unique CHD8 binding sites at promoters. Transcription factor ChIP-seq experiments in the UniBind database are ranked on the X axis and ChIP-seq binding patterns that overlap are colored according to the indicated corresponding transcription factor. Overlap significance threshold and –log10(*p*-value) of the overlap are indicated on the Y axis. (I) Enrichment of transcription factor binding sites at unique TRRAP binding sites at promoters. Transcription factor ChIP-seq experiments in the UniBind database are ranked on the X axis and ChIP-seq binding patterns that overlap are colored according to the indicated corresponding transcription factor. Overlap significance threshold and –log10(*p*-value) of the overlap are indicated on the Y axis.

Gene set enrichment analyses (GSEA) of hNSC promoters bound by overlapping CHD8 and TRRAP showed high enrichments of MYC and E2F target genes (Figure 3A). Interestingly, CHD8 and TRRAP sites that do not overlap show no enrichment (unique CHD8 promoters) or less enrichment (unique TRRAP promoters) of MYC and E2F target genes (Figures 3B and 3C). This may suggest that CHD8 and TRRAP specifically bind together on MYC and E2F target gene promoters. We used the UniBind transcription factor binding site enrichment tool, which includes 1983 ChIP-seq datasets for 232 human transcription factors,^39^ to determine enriched transcription binding sites at our CHD8 and TRRAP bound regions. We found that genome-wide sites where CHD8 and TRRAP colocalized showed the highest enrichments for MYC, MYCN, BACH and E2F1 binding sites (Figure 3D). CHD8 sites and TRRAP sites that did not overlap (unique sites) showed no enrichment for MYC or E2F binding sites (Figures 3E and 3F). CHD8-TRRAP overlapping sites at promoters were strongly enriched for MXI1, MYC, E2F1, E2F4 and MAX (Figure 3G). MXI1 is a repressor that competes with MYC for MYC binding sites and for binding to MYC-heterodimer partner and co-activator MAX.^40^^,^^41^^,^^42^ E2Fs strongly colocalize in their genome-wide binding with MYC.^43^^,^^44^ Binding sites for MYC and its binding partners and E2F transcription factors were not enriched at non-overlapping CHD8 and TRRAP sites at promoters (Figures 3H and 3I). Together, we find that CHD8 and TRRAP often coincide at promoter sites containing MYC and E2F binding sites and at promoters of MYC and E2F target genes.

### CHD8 and TRRAP maintain expression of MYC and E2F target genes

**Figure 4:**
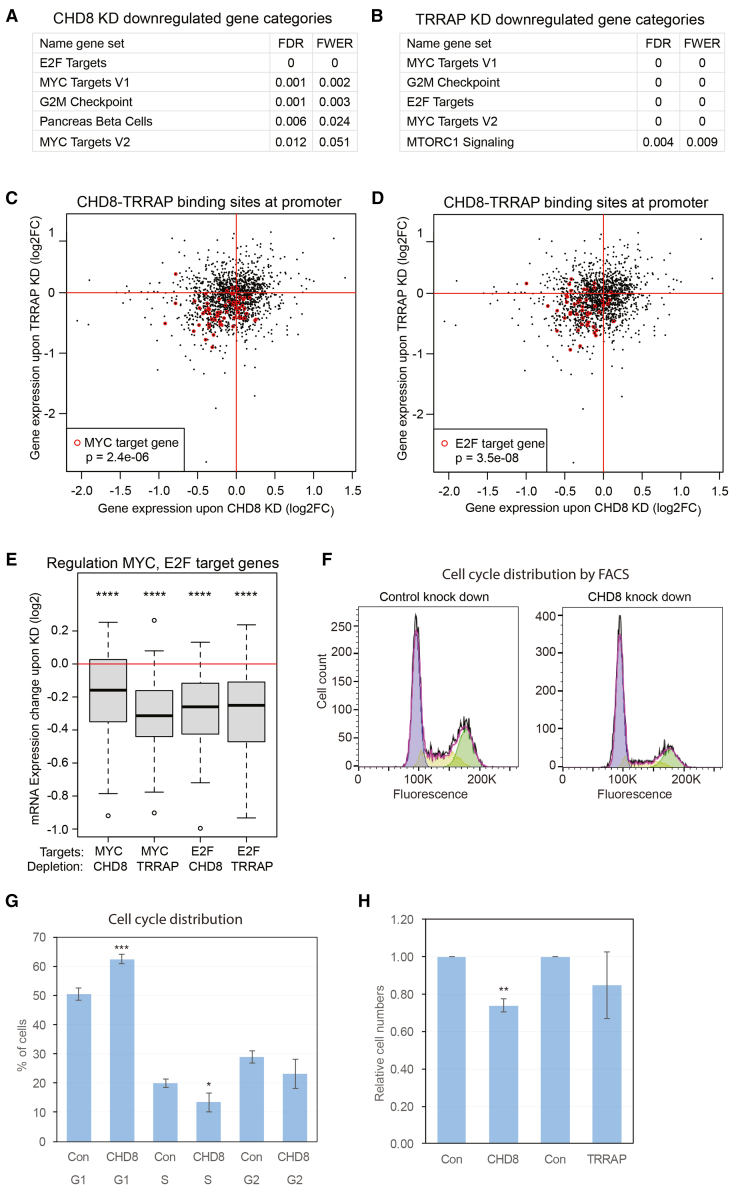
CHD8 and TRRAP maintain expression of MYC and E2F target genes (A) Gene Set enrichments for downregulated genes upon knockdown of CHD8. False discovery rate q-value (FDR) and family-wise error rate *p*-value (FWER) are indicated. (B) Gene Set enrichments for downregulated genes upon knockdown of TRRAP. False discovery rate q-value (FDR) and family-wise error rate *p*-value (FWER) are indicated. (C) Scatterplot showing changes in expression of genes that are promoter-bound by overlapping CHD8 and TRRAP, upon depletion of either CHD8 (X axis) or TRRAP (Y axis). Gene expression changes are indicated as log2FC. MYC target genes are marked with red circles. The depicted *p*-value (Fisher exact test) is an indication of enrichment of MYC target genes in the quadrant where the genes are downregulated upon knockdown of CHD8 and knockdown of TRRAP. (D) Scatterplot showing changes in expression of genes that are promoter-bound by overlapping CHD8 and TRRAP, upon depletion of either CHD8 (X axis) or TRRAP (Y axis). Gene expression changes are indicated as log2FC. E2F target genes are marked with red circles. The depicted *p*-value (Fisher exact test) is an indication of enrichment of E2F target genes in the quadrant where the genes are downregulated upon knockdown of CHD8 and knock down of TRRAP. (E) Boxplot showing expression levels of MYC target genes and E2F target genes upon depletion of CHD8 or TRRAP. The change in mRNA expression upon knockdown (log2) is depicted on the y axis. Expression levels of MYC target genes are significantly lowered (Wilcoxon test) upon CHD8 knockdown (*p*-value = 6 e^−6^) or upon TRRAP knockdown (*p*-value = 2 e^−10^). The expression levels of E2F target genes are also significantly reduced upon CHD8 knockdown (*p*-value = 1.7 e^−11^) or upon TRRAP knockdown (*p*-value = 1.6 e^−8^). P-values <0.0001 are indicated with ∗∗∗∗. (F) Propidium iodine (PI) FACS of hNSCs with knockdown of CHD8 (right panel) or control knockdown (left panel). Typical cell count distribution on PI fluorescence is shown. Different colorings indicate cells populations in G1 phase, S phase and G2 phase. (G) Percentages of cells in G1, S and G2 phase. CHD8 indicates hNSCs with knock down CHD8, Con indicates hNSCs with control knock down. Average of 4 biological replicate experiments and standard deviation is shown. P-values (two-tailed t-test) of differences between Con and CHD8 are shown; P-value <0.001 is indicated with ∗∗∗ and P-values <0.05 is indicated with ∗. (H) Relative cell numbers after 72 h knockdown. CHD8 indicates hNSCs with knockdown CHD8, TRRAP indicates hNSCs with knockdown TRRAP, Con indicates hNSCs with control knockdowns for CHD8 (left) and TRRAP (right), respectively. Average of 4 biological replicate experiments and standard deviation is shown. P-value (two-tailed t-test) of difference between Con and CHD8 is shown; P-value <0.01 is indicated with ∗∗.

To assess a potential role of CHD8 and TRRAP in gene regulation in hNSCs, we performed shRNA-mediated knockdowns for *CHD8* or *TRRAP* and performed RNA-sequencing after 72 h. Knockdown of *CHD8* for 72 h reduced *CHD8* mRNA levels to 15% compared to hNSCs transfected with control shRNA (Figure S1C). Depletion of *TRRAP* for 72 h decreased *TRRAP* mRNA levels to 33% (Figure S1D). Genes that were up- or downregulated upon knockdown of *CHD8* or *TRRAP*, were identified by RNA-seq analysis (Table S3). Gene set enrichment analyses (GSEA) on downregulated genes upon knockdown of either *CHD8* or *TRRAP* showed MYC and E2F target genes as most enriched gene sets (Figures 4A and 4B). Out of 200 MYC target genes, 115 genes were significantly (FDR<0.05) downregulated upon knockdown of *CHD8* and 115 MYC target genes were downregulated upon TRRAP depletion (Table S3). Out of 199 E2F target genes, 139 genes and 95 genes were significantly downregulated upon knockdown of *CHD8* or *TRRAP*, respectively (Table S3). *MYC* itself is modestly downregulated to 67% of control levels upon depletion of CHD8 (Table S3) but 3-fold upregulated upon *TRRAP* knockdown (Table S3). The knockdown of CHD8 or TRRAP has at most modest effects on the expression of genes for the E2F transcription factors, from no effect to downregulation to 60% of control levels (Table S3). MYC and E2F target genes, bound at their promoter by overlapping CHD8 and TRRAP, are predominantly downregulated upon knockdown of *CHD8* or *TRRAP* (Figures 4C and 4D). Furthermore, the expression levels of MYC and E2F target genes decreased upon depletion of either CHD8 or TRRAP (Figure 4E). MYC knockdown in hNSCs (Figure S1E) showed downregulation of most GSEA-assigned MYC target genes and a significant (FDR<0.05) downregulation of 31 genes (Figure S1F) indicating that GSEA-assigned MYC target genes are also regulated by MYC in hNSCs. These data suggest that CHD8 and TRRAP are required for maintaining the expression of MYC and E2F target genes in hNSCs.

### CHD8 depletion diminishes cell cycle entry into S-phase and proliferation

As E2F and MYC target genes regulate entry into S-phase,^45^^,^^46^ we assessed whether depletion of CHD8 or TRRAP from hNSCs affected their cell cycle profile by FACS on DNA content. Depletion of CHD8 increased the fraction of cells in G1 and decreased the fraction in S-phase (Figures 4F and 4G), suggesting a diminished entry into S-phase. Depletion of TRRAP leads to a block in G2-M, as is known from literature^47^^,^^48^ (Figures S2A and S2B). Depletion of CHD8 decreased proliferation of hNSCs, as measured by cell count (Figure 4H).

## Discussion

CHD8 was shown previously to bind and activate E2F target genes in immortalized cell lines upon entry of the cell cycle or S-phase.^26^ In these cells, CHD8 directly interacts with E2F1 and is necessary for E2F1 loading onto several promoters of G1/S transition-induced genes.^26^ *Chd8* knockdown in mouse NSCs *in utero* decreases the proliferation speed and downregulates the expression of cell cycle genes.^14^ Likewise, the genetic deletion of *Trrap* causes lengthening of the cell cycle and downregulation of the E2F target gene expression in *ex vivo* mouse NSCs.^32^ This decreased gene expression of E2F-dependent cell cycle genes is caused by a reduced loading of E2F1 on their promoters.^32^ Moreover, the TRRAP complex is a well-known co-activator of MYC and E2F transcription factors.^30^ Together, this suggests that both CHD8 and TRRAP are important for NSC proliferation in mice and that they may do so by maintaining expression of E2F target genes.

Here, we demonstrate that these activities of CHD8 and TRRAP are connected in hNSCs. We show that CHD8 and TRRAP proteins physically interact and that they colocalize genome-wide. Interestingly, their colocalization on the genome is strongest at E2F and MYC target genes and neither CHD8 nor TRRAP binds alone to promoters of E2F or MYC target genes. This suggests that TRRAP and CHD8 act as a transcriptional activation unit to help MYC and E2F activate their target genes in hNSCs. As both TRRAP and CHD8 were shown separately to facilitate binding of E2F1 to promoters,^26^^,^^32^ this may also be the mode of action by which a CHD8-TRRAP complex facilitates gene activation by E2F. We show that CHD8 depletion causes diminished entry into S-phase, which matches a role for CHD8 in activating E2F and MYC target genes. TRRAP as an MYC co-activator was suggested to act in a different manner. A recent study shows that degradation of MYC is essential for its role in transcriptional activation.^49^ A ubiquitination-resistant form of MYC binds to its genomic target sites normally but cannot recruit TRRAP, BRD4 and CDK9 and therefore cannot activate RNA polymerase II, leading to transcriptional inhibition.^49^ This suggests that TRRAP is not involved in the recruitment of MYC to its target sites but in the subsequent activation of transcription by MYC. We did observe BRD4 in one of our CHD8 purifications. CHD8 was previously shown to bind BRD4 via NSD3 in leukemia cells and act as a functional CHD8-NSD3-BRD4 unit in transcriptional activation.^29^ The CHD8-BRD4 interaction was also observed in two high-throughput interaction studies.^50^^,^^51^ Intriguingly, we observed MYCBP2 as an interactor of CHD8 (Table S1). MYCBP2 binds and ubiquitinates MYC, leading to MYC protein degradation.^52^^,^^53^ Therefore, recruitment of CHD8-TRRAP by MYC may facilitate both recruitment of BRD4 and ubiquitination of MYC by MYCBP2, thus enabling the rapid transcription activation/degradation cycle of MYC. Our study thereby bridges functional interactions observed in other studies and suggests that CHD8, via its interactions with TRRAP, BRD4, and MYCBP2, participates in transcriptional activation by MYC. The promoter of the *MYC* gene is significantly bound by CHD8 but not TRRAP, is modestly downregulated by CHD8 knockdown and upregulated by TRRAP knockdown. This may suggest that CHD8 is a weak but direct regulator of *MYC*. *MYC* upregulation upon TRRAP knock down may be an indirect compensatory mechanism for less effective activation of MYC target genes by MYC without its cofactor TRRAP.

### Limitations of the study

Although the physical interaction of CHD8 and TRRAP, their co-localization on the promoters of E2F and MYC target genes and their requirement for maintaining their expression suggest cooperativity between CHD8 and TRRAP in the regulation of E2F and MYC target genes, our experiments do not prove that the interaction between CHD8 and TRRAP is necessary for this regulation. Experiments that do prove the necessity of the interaction for regulating MYC and E2F target genes are impossible as (1) they require the knowledge of the interaction surface of CHD8 with the TRRAP complex and (2) proof that this interaction surface is not required for other important interactions for the regulation of MYC and E2F target genes. Whereas (1) can be achieved at great cost by interaction studies of parts of CHD8 with parts of subunits of the TRRAP complex, (2) is impossible to achieve as one can never rule out that the identified interaction surface is also used by other (unknown) proteins that affect the regulation of MYC and E2F target genes.

## Resource availability

### Lead contact

Further information and requests for resources and reagents should be directed to and will be fulfilled by the lead contact, Raymond Poot (r.poot@erasmusmc.nl).

### Materials availability

All unique/stable reagents generated in this study are available from the lead contact.

### Data and code availability

• The genomics data reported in this paper were submitted to GEO database under accession number: GSE247133 (CHD8 ChIP-seq bigwig, TRRAP ChIP-seq bigwig, IgG ChIP-seq bigwig, RNA-seq KD CHD8, KD TRRAP, scrambled, KD MYC, scrambled). The mass spectrometry proteomics data have been deposited to the ProteomeXchange Consortium via de PRIDE partner^54^ with the dataset identifier PXD044582 (CHD8, control, 3 exps).
• This paper does not report original code.
• This paper does not contain other items.

## Acknowledgments

M. P de A. was supported by an ALW-open program grant (ALWOP.372) from NWO.

## Author contributions

L.M. generated hNSC lines, performed CHD8 purifications, knockdown experiments, and CHD8 and TRRAP ChIP-seq, M.P.d.A. performed knockdown experiments and V5-ChIP, M.R.D. performed CHD8 purifications, immunoprecipitations, and knockdown experiments, D.H.W.D. and J.D. performed mass spectrometry analyses, K.N. performed bioinformatic analyses on ChIP-seq data, D.H. provided scientific input, M.v.d.H., Z.O., and W.F.J.v.IJ. performed RNA-seq and ChIP-seq, M.F. performed bioinformatics analyses on RNA-seq and ChIP-seq data, R.A.P. conceived and supervised the project and wrote the manuscript with help from co-authors.

## Declaration of interests

R.A.P. is on the advisory board of *Cell Reports*. The authors have no other competing interests.

## STAR★Methods

### Key resources table

**Table:** 

REAGENT or RESOURCE	SOURCE	IDENTIFIER
**Antibodies**		

Mouse monoclonal Anti-FLAG	Sigma-Aldrich	Cat# F3165; RRID: AB_259529
Rabbit polyclonal Anti-CHD8	Bethyl	Cat# A301-224A; RRID: AB_890578
Goat anti-Mouse IgG-heavy and light chain Antibody HRP Conjugated	Bethyl	Cat# A90-116P; RRID: AB_67183
Goat anti-Rabbit IgG-heavy and light chain Antibody HRP Conjugated	Bethyl	Cat# A120-101P; RRID: AB_67264
Mouse anti-Actin	Millipore	Cat# MAB1501; RRID: AB_2223041
Mouse monoclonal Anti-FLAG M2 Affinity Gel	Sigma-Aldrich	Cat# A2220; RRID: AB_10063035
Rabbit polyclonal Anti-TRRAP	Invitrogen	Cat# PA5-78246; RRID: AB_2736753
Dynabeads Protein G	Invitrogen	Cat# 10004D
Rabbit-IgG	Diagenode	Cat# C15410206; RRID: AB_2722554
Rabbit anti-CHD8	Bethyl	Cat# A301-225A; RRID: AB_890577
Mouse monoclonal Anti-V5 Agarose Affinity Gel	Sigma-Aldrich	Cat# A7345 RRID: AB_10062721

**Bacterial and virus strains**		

DH5 alpha Competent Cells	Invitrogen	Cat# 18265017

**Chemicals, peptides, and recombinant proteins**		

Geltrex LDEV-free membrane matrix	Thermo Fisher Scientific	Cat# A1413202
EGF	Peprotech	Cat# 315-09
FGF	Peprotech	Cat# 100-18B
StemPro Neural Supplement	Thermo Fisher Scientific	Cat# A1050801
Accutase	Sigma-Aldrich	Cat# A6964
Bbs I	New England Biolabs	Cat# R0539
T4 DNA ligase	Promega	Cat# M1801
Cell Line Nucleofector™ Kit V	Lonza Biosystems	Cat# VCA-1003
Puromycin	Sigma-Aldrich	Cat# P8833
cOmplete, EDTA-free Protease Inhibitor Cocktail	Roche	Cat# 11873580001
Benzonase	Novagen	Cat# 70664
3xFLAG Peptide	Sigma-Aldrich	Cat# F4799
DSG crosslinker	Thermo Fisher Scientific	Cat# 20593
Formaldehyde	Sigma-Aldrich	Cat# 252549
SYBR Green I nucleic acid gel stain	Sigma-Aldrich	Cat# S9430

**Critical commercial assays**		

NucleoBond Xtra Midi	Macherey-Nagel	Cat# 740410
GenElute Mammalian Total RNA Miniprep Kit	Sigma-Aldrich	Cat# RTN350-1KT
RevertAid First Strand cDNA Synthesis Kit	Fermentas	Cat# K1621
TruSEq Stranded mRNA library Prep kit	Illumina	Cat# 20020595

**Deposited data**		

The genomics data reported in this paper	GEO database	GSE247133
The mass spectrometry proteomics data reported in this paper	ProteomeXchange Consortium via de PRIDE partner	PXD044582
Human reference genome NCBI build 38, GRCh38	Genome Reference Consortium	https://www.ncbi.nlm.nih.gov/datasets/genome/GCF_000001405.26/
Uniprot human database, release 2017-04	Uniprot	https://www.uniprot.org/
Transcription Factor motifs UniBind database	Puig et al.^39^	https://unibind.uio.no/
HALLMARK_MYC_TARGETS_V1	GSEA	https://www.gsea-msigdb.org/gsea/msigdb/human/collections.jsp
HALLMARK_E2F_TARGETS	GSEA	https://www.gsea-msigdb.org/gsea/msigdb/human/collections.jsp

**Experimental models: Cell lines**		

Human: H9 Neural stem cells	Invitrogen	Cat# N7800-100

**Oligonucleotides**		

gRNA: C-terminus CHD8: AAGCAATGGGGCCCATGCTG	This paper	N/A
shRNA targeting sequence: CHD8: GAACTACTCCTATCTGCAT	This paper	N/A
shRNA targeting sequence: C-MYC: GGACTATCCTGCTGCCAAG	Feng et al.^55^	N/A
shRNA targeting sequence: control: GGTGAGCTTCATGAGGATG	Dharmacon	N/A
shRNA targeting sequence: MISSION® TRC-Hs 1.5 (Human) TRRAP: GCCCTGTTCTTTCGCTTTGTA	Sigma-Aldrich	N/A

**Recombinant DNA**		

pSpCas9(BB)-2A-Puro (PX459)	Addgene	Cat# 62988
pSuper-puro plasmid	Oligoengine	Cat# VEC-pBS-0008
PLKO.1-puro	Sigma-Aldrich	Cat# SHC001
MISSION pLKO.1-puro Non-Mammalian shRNA	Sigma-Aldrich	Cat# SHC002

**Software and algorithms**		

MASCOT search engine v2.3	Matrix Science	https://www.matrixscience.com/daemon_support_v2_3.html
BioGRID	BioGRID	https://thebiogrid.org/
MaxQuant software suite v2.2.0.0	MaxQuant	https://www.maxquant.org/
HISAT2	Kim et al.^56^	https://daehwankimlab.github.io/hisat2/
MACS2 v2.1.1.20160309	Zhang et al.^57^	https://github.com/macs3-project/MACS
ENCODE Blacklist	Amemiya et al.^58^	https://github.com/Boyle-Lab/Blacklist/
GenomicRanges package v.1.48	Lawrence et al.^59^	https://bioconductor.org/packages/release/bioc/html/GenomicRanges.html
ChiPseeker v1.18.0	Bioconductor	https://www.bioconductor.org/packages/devel/bioc/vignettes/ChIPseeker/inst/doc/ChIPseeker.html
GSEA 4.1.0	Subramanian et al.^60^	https://www.gsea-msigdb.org/gsea/downloads.jsp
EdgeR	Robinson et al.^61^	https://bioconductor.org/packages/release/bioc/html/edgeR.html
UniBind Enrichment Tool	Puig et al.^39^	https://unibind.uio.no/enrichment/

**Other**		

Amaxa Nucleofector I	Lonza Biosystems	N/A
Bioruptor Pico sonication device	Diagenode	Cat# B01060001
HiSeq2500 sequencer	Illumina	N/A

### Experimental model and study participant details

#### Human neural stem cells

H9 human embryonic stem cell-derived neural stem cells were purchased from Invitrogen (N7800-100). hNSCs were cultured on Geltrex-coated dishes (Geltrex™ LDEV-free reduced growth factor membrane matrix, A1413202, Thermo Fisher Scientific) in KnockOut™ DMEM/F12 (12660012, Invitrogen) supplemented with 2 mM L-Glutamine (25030024, Thermo Fisher Scientific), 20 ng/ml EGF (315-09, Peprotech), 20 ng/ml FGF (100-18B, Peprotech) and 2% of StemPro® Neural Supplement (A1050801, Thermo Fisher Scientific). The media was refreshed every other day and the cells were routinely passaged with Accutase (A6964, Sigma-Aldrich). The hNSC cultures were checked for mycoplasma contamination every three months.

### Method details

#### CRISPR-Cas9-mediated insertion of a double Flag V5 tag at the C-terminus of CHD8

The gRNA (5’-AAGCAATGGGGCCCATGCTG-3’) targeting the C-terminus of CHD8 was designed using crispr.mit.edu and cloned in the pSpCas9(BB)-2A-Puro plasmid (PX459, Addgene), using the BbsI restriction enzyme (R0539, NEB) and T4 ligase (M1801, Promega). For amplifying the plasmid, DH5α competent bacteria (#18265017, Invitrogen) were used, after the DNA was isolated using a midiprep kit (740410, Macherey Nagel). To integrate a double Flag-tag and a single V5-tag at the C-terminal end of the CHD8 protein, we designed a single-stranded DNA-oligo of 200bp containing the sequence for a double Flag tag, single V5-tag flanked by approximately 50bp of genomic homology arms to use as a homology template. hNSCs (3.5x10^6^) were transfected with 3 μg of PX459 plasmid containing the gRNA and 1 μg of the DNA-oligo by electroporation with the Amaxa nucleofector I using the Cell Line Nucleofector™ Kit V (VCA-1003, Lonza) and program A33. After transfection, the cells were plated at clonal density and were allowed to recover. 24hrs after transfection, puromycin (P8833, Thermo Fisher) selection was added at a concentration of 1μg/ml for 48hrs and transfected cells started to form colonies. The media was refreshed every other day, until the colonies were picked, expanded and genotyped by PCR. The resulting candidate hNSC F-CHD8 clones were validated using sanger sequencing and by checking the expression of the Flag-tagged CHD8 protein by western blot. In brief, the cells were harvested and lysed with 2x Laemmli sample buffer (0.2M DTT, 4% SDS, 1M Tris HCl pH 6.8, 20% Glycerol). After sonication, the protein samples were run on a NuPAGE 4 to 12% Bis-Tris gel (M41215, GenScript). Gels were then transferred onto nitrocellulose membranes (Amersham Bioscience). The membranes were blocked in 5% Fat-free milk proteins in TBS 0,1% Tween and probed overnight with Flag antibody (Sigma-Aldrich, F3165, 1:1000) and CHD8 antibody (Bethyl A301-224A, 1:1000) at 4°C. Probed blots were incubated at RT with HorseRadish Peroxidase (HRP) conjugated secondary anti-mouse (A90-116P, Bethyl) and anti-Rabbit (A120-101P, Bethyl) antibodies, respectively. The protein bands were visualized on an AI-600 digital imager (Amersham). As loading control, an anti-Actin antibody (MAB1501R, Chemicon) was used.

#### Flag affinity purification of CHD8 followed by mass spectrometry

Nuclear extracts were made of hNSCs expressing Flag-tagged CHD8 and from control hNSCs, according to the well-established Dignam protocol.^62^ To identify interaction partners of the CHD8 protein in hNSCs, FLAG-affinity purifications were performed on 1.5 ml of dialyzed nuclear extract and analyzed by mass spectrometry, as described previously.^35^^,^^36^ In brief, 60 μl of anti-FLAG M2 affinity agarose beads (Sigma-Aldrich) were equilibrated in buffer C-100∗ (20 mM Hepes pH 7.6, 0.2 mM EDTA, 1.5 mM MgCl_2_, 100 mM KCl, 20% Glycerol, 0.02% NP40 and 1x CEF protease inhibitor (Roche)) and added to 1.5 ml of nuclear extract in no-stick microcentrifuge tubes (Alpha Laboratories). The mixture of beads and nuclear extract was rotated at 4°C for 3hrs in the presence of 225 units of Benzonase (#70664, Novagen). After incubation, the beads were washed sequentially 5 times with buffer C-100∗. The bound proteins were then eluted off the beads at 4°C by 4 subsequent washes with buffer C-100∗ containing 0.2 mg/ml of FLAG-tripeptide (F4799, Sigma-Aldrich). TCA precipitation was performed on the elutions. The precipitated proteins were then separated on a 10% NuPAGE Novex Bis-Tris gel (NP0301, Invitrogen) and stained with the Colloidal Coomassie Blue Staining Kit (LC6025, Invitrogen). The gel lanes were cut into slices and subjected to in-gel digestion with trypsin (TPCK-treated, #20233, Thermo Scientific™). Nanoflow LC-MS/MS was performed using 60 min gradients on an EASY-nLC 1000 Liquid Chromatograph (Thermo Scientific™) coupled to an Orbitrap Fusion™ Tribrid™ Mass Spectrometer (Thermo Scientific™). The resulting peptide spectra from the F-CHD8 or the control elutions were then identified by MASCOT search engine (Matrix Science, v2.3) using the Uniprot human database (release 2017-04, including isoforms). The following inclusion criteria for the CHD8 interaction partners were used; a MASCOT score of 50 or higher, at least 3-fold enrichment of EmPAI score in the CHD8 purified sample compared to the control elution and only nuclear proteins were considered (based on the Uniprot database). In total three independent nuclear extract preparations and flag affinity purifications were performed from F-CHD8 NSCS and from control hNSCs. Interaction partners that were identified in 3 out of 3 or 2 out of 3 experiments were included in the final list of identified CHD8 interactors. The BioGrid database was used to verify the novel interactions.^63^ Raw mass spectrometry data were analyzed using the MaxQuant software suite [version 2.2.0.0^64^] for identification and relative quantification of proteins. “Match between runs” was disabled, and a false discovery rate (FDR) of 0.05 for proteins and peptides and a minimum peptide length of six amino acids were required.

#### Immunoprecipitations

For TRRAP and CHD8 immunoprecipitations, 250 μl of NSC nuclear extract with 38 units of Benzonase (Novagen) and 1x CEF protease inhibitor (Roche) were rotated with 3 μg TRRAP antibody (Invitrogen PA5-78246) or 3 μg of CHD8 antibody (Bethyl A301-224A) for 2 hours at 4°C in no-stick microcentrifuge tubes (Alpha Laboratories). 40 μl of ProtG Dynabeads (Invitrogen, #100-04D) equilibrated with 1 ml of C-100 buffer (20 mM Hepes pH 7.6, 0.2 mM EDTA, 1.5 mM MgCl_2_, 100 mM KCl, 20% glycerol) were blocked with 0.1 mg/ml insulin (Sigma-Aldrich) and 0.2 mg/ml chicken egg albumin (Sigma-Aldrich) in C-100 buffer for 1 hour rotating at room temperature and washed twice with C-100 buffer. Incubated hNSC nuclear extract with antibody was added to the blocked Dynabeads and rotated for 2 hours at 4°C in no-stick microcentrifuge tubes. Bound proteins were eluted from the beads by incubation at 95°C for 5 minutes in 28,5 μl 2x Laemmli sample buffer (0.2M DTT, 4% SDS, 1M Tris HCl pH 6.8, 20% Glycerol). As a control, an IgG (Diagenode C15410206) immunoprecipitation was performed in the same manner. The elution samples were run on a 6% SDS-PAGE gel. Resulting gels were transferred on PVDF membranes (Millipore Immobilon-P). The membranes were blocked in 5% Fat-free milk proteins in TBS 0,1% Tween and probed overnight with TRRAP antibody (Invitrogen PA5-78246, 1:1000) and CHD8 antibody (Bethyl A301-224A, 1:1000), followed by incubation at RT with HorseRadish Peroxidase (HRP) conjugated secondary anti-Rabbit antibody (A120-101P, Bethyl). The protein bands were visualized on an AI-600 digital imager (Amersham).

#### Chromatin immunoprecipitation (ChIP)

Chromatin immunoprecipitations were performed as previously described.^65^ In brief, hNSCs were collected in 1x PBS + 1mM PMSF and double crosslinked by incubation with 2 mM disuccinimidyl glutarate (#20593, Thermo Fisher Scientific) solution, rotating at room temperature for 45min, followed by an incubation with 1% of buffered formaldehyde (#252549, Sigma-Aldrich) solution for 10min at room temperature. Subsequently, the cells were washed twice with ice-cold 1X PBS + 1mM PMSF and flash frozen in liquid nitrogen. The cell pellets were lysed by adding 4 volumes of lysis buffer (1% SDS, 50 mM TrisHCl pH 8.1, 10mM EDTA pH 8.0 and 1x CEF protease inhibitors (Roche)) and sonicated using a Bioruptor Pico sonication device (Diagenode, B01060001) for 14 cycles of 30sec on and 30sec off. The ChIP was performed on the resulting chromatin (300 μg) by using 100 μl of ProtG Dynabeads (Invitrogen, #100-04D) coupled to either 10 μg of CHD8 antibody (Bethyl, A301-225A), 20 μg of TRRAP antibody (Invitrogen, PA5-78246) or IgG antibody (Diagenode, C15410206) as control. First the Dynabeads were equilibrated in ChIP dilution buffer (0.01% SDS, 1.1% Triton X-100, 1.2 mM EDTA, 16.7 mM TrisHCL pH 8.1, 167 mM NaCl) and coupled to the corresponding antibody diluted in 1x PBS + 0.02% Tween by rotation for 3 hours at room temperature. After incubation, the Dynabeads were sequentially washed with 1x PBS + 0.02% Tween and ChIP dilution buffer. The sonicated chromatin was added an incubated rotating overnight at 4°C. The Dynabeads were then washed twice with Low salt wash buffer (20 mM Tris-HCl pH 8.0, 2 mM EDTA, 1% Triton X-100, 150 mM NaCl), once with High salt wash buffer (20 mM Tris-HCl pH 8.0, 2 mM EDTA, 1% Triton X-100, 500 mM NaCl), once with LiCl wash buffer (10 mM Tris-HCl pH 8.0, 1 mM EDTA, 0.25 M LiCl, 0.5% IGEPAL, 1% NaDeoxycholate) and once with T_10_E_1_ (10 mM TrisHCl pH 8.0, 1 mM EDTA). Then, the bound complexes were eluted of the beads by incubation for 1 hour at 65°C in ChIP elution buffer (50 mM Tris-HCl pH 7.5, 10 mM EDTA, 1% SDS) with occasional gentle vortexing. The samples were de-crosslinked overnight by incubation at 55°C. DNA was extracted from the elutions by the PCI method and diluted in water. CHD8-V5 ChIP was performed as described above but using anti-V5 antibody agarose beads (Sigma, A7345) and using wild-type hNSCs for the control anti V5-ChIP.

#### ChIP-seq

The DNA libraries for the CHD8, TRRAP and IgG ChIPs were prepared using the ThruPLEX V2 DNA sample preparation protocol from Takara Bio and sequenced on an Illumina HiSeq2500 sequencer at the Erasmus MC Center for Biomics. Single reads of 50 bp in length were generated. The reads were first trimmed by removing the Illumina adapters, followed by alignment to the reference genome GRCh38 using HISAT2.^56^ After alignment, low-quality, duplicated fragments and fragments that exceed 150 bases in length were removed.

#### Knockdown experiments in hNSCs

CHD8 short hairpin RNA (5’-GAACTACTCCTATCTGCAT-3’) and C-MYC shRNA (5’-GGACTATCCTGCTGCCAAG-3’)^55^ (Feng et al., 2020) sequences were cloned into the pSuper-puro construct (Oligoengine). hNSCs (3.5x10^6^) were transfected with 3.5 μg of each construct by electroporation using the Cell Line Nucleofector™ Kit V (Lonza, catalog # VVCA-1003) on the Amaxa nucleofector I (program A-33) and plated on a 10-cm dish. As a control, pSuper-puro-Control-shRNA (scrambled control sequence 5’- GGTGAGCTTCATGAGGATG-3’ from Dharmacon) transfections were performed in the same manner. For the TRRAP shRNA-mediated knockdown, the shRNA (5’- GCCCTGTTCTTTCGCTTTGTA-3’, Sigma Mission® TRC-Hs 1.5 library) was cloned into the PLKO.1-puro plasmid. The hNSCs (3.5x10^6^) were transfected with 7 μg of plasmid as described above. A PLKO.1-puro-Control-shRNA (MISSION® pLKO.1-puro Non-Mammalian shRNA, SHC002, 5’- CAACAAGATGAAGAGCACCAA-3’, Sigma-Aldrich) was used to perform the control experiments in the same manner. For all knockdown experiments, selection was started 24 hours after transfection with 1 μg/ml Puromycin. The transfected hNSCs were collected for RNA isolation after 48 hours of selection (72 hours after transfection).

RNA was isolated using the GenElute™ Mammalian Total RNA Miniprep Kit (Sigma-Aldrich, RTN350-1KT) and reverted to cDNA using RevertAid First Strand cDNA Synthesis Kit with OligodT-primers (Fermentas, K1621). RT-qPCR was performed using SYBRGreen (S9430, Sigma-Aldrich) on a CFX96 T1000 thermal cycler (BioRad) to determine the knockdown efficiency. Three separate transfections, RNA isolations and RNA-seq were performed per condition.

#### RNA-seq

The total RNA was prepped using the Illumina TruSEq Stranded mRNA library Prep kit and sequenced according to the Illumina TruSeq Rapid v2 protocol on an HiSeq2500 sequencer (Illumina) at the Erasmus MC Center for Biomics. Reads were generated of 50 bp in length. First, the Illumina adapters and poly-A-sequences were trimmed of the reads, followed by alignment to GRCh38 using HISAT2.^56^ Last, quantification of the reads was performed using HT-seq count.^66^

#### Data analysis

For ChIP-seq analyses, peak calling, MACS2 (version 2.1.1.20160309)^57^ was used with FDR thresholds 1e-4 (CHD8) and 1e-3 (TRRAP). Peaks overlapping with ENCODE Blacklist^58^ have been removed using GenomicRanges package v.1.48 in R.^59^ Subsequently, the peaks longer than 1kb have been filtered as they are mostly artefacts not presenting narrow peaks for TF binding. Overlapping and individual (unique) CHD8 and TRRAP peaks have been identified using ‘findOverlaps’ function from GenomicRanges.^59^ Peak annotation was done with ChiPseeker (Bioconductor, version 1.18.0) using a 1 kb window from annotated TSS as promoter. P values of overlaps of binding sites (Figures 2B, 2D, and S1B) were calculated using a hypergeometric test with the number of possible binding places equal the sum all detected peaks. This probably underestimates the real number of possible binding places and therefore overestimates the p values - however, these are approaching zero. Genes associated with promoters with CHD8-TRRAP binding sites, unique CHD8 binding sites or TRRAP unique binding sites were analyzed for enriched gene sets by Gene Set Enrichment Analyses using default Hallmark gene sets^60^ (GSEA 4.1.0).

Differential gene expression was calculated using edgeR^61^ and ranked values were employed in Gene Set Enrichment Analyses using default Hallmark gene sets^60^ (GSEA 4.1.0). TF motif enrichment analysis for defined subsets of individual or overlapping CHD8 and TRRAP peaks have been performed using UniBind Enrichment Tool^39^ (https://unibind.uio.no/enrichment/) with a background defined as all CHD8 and TRRAP binding regions. TF motifs from the UniBind database,^39^ which are defined based on ChIP-seq experiments, have been used for the enrichment analysis. For association of differential gene expression (72h post RNAi) and promoter binding with MYC knockdown differential gene expression or target binding, a log2 expression intensity (CPM) CHD8 or TRRAP cutoff was used of greater than 5. MYC and E2F binding targets were taken from Hallmark gene sets HALLMARK_MYC_TARGETS_V1 and HALLMARK_E2F_TARGETS (GSEA).

### Quantification and statistical analysis

Significance overlap binding sites CHD8 and TRRAP (Figures 2B, 2D, and S2B) was calculated by hypergeometric test. Significance differences in expression in genes with different promoter types (Figure 2E) was calculated by Wilcoxon test. Gene set enrichment significances (Figures 3A–3C, 4A, and 4B are indicates by false discovery rate q-value (FDR) and family-wise error rate p-value (FWER). Enrichment MYC targets (Figure 4C) or E2F targets (Figure 4D) is by Fisher exact test. Significance of changes in gene expression MYC or E2F target genes upon depletion of CHD8 or TRRAP in triplicate (Figure 4E) is by Wilcoxon test. Significance of differences in cell cycle phase between CHD8 knock down and control or TRRAP knockdown and control (Figure 4G) or TRRAP knockdown and control (Figure S2B), 4 biological replicates, by two-tailed t-test. Significance of differences in cell numbers between CHD8 knockdown and control or TRRAP knockdown and control (Figure 4H) , 4 biological replicates, by two-tailed t-test.

## Supplemental information

Document S1. Figures S1 and S2

Table S1. Mass spectrometry results of F-CHD8 protein purifications Results of 3 independent purifications (IP #1, IP #2, IP #3) and their average are shown. For CHD8 and identified interaction partners, number of identified unique peptides, Mascot score and EmPAI scores are shown for CHD8 and the corresponding control purifications (WT Control) are shown.

Table S2. MaxQuant analyses of mass spectrometry on 3 F-CHD8 protein and control purifications (# 1, #2, #3) TRRAP complex subunits are highlighted. CHD8 in red. Only proteins detected in CHD8 vs. control samples with an FDR <0.05 are shown.

Table S3. Gene expression changes upon knockdowns Log Fold Change (logFC), LogCPM, P-values, MYC E2F HALLMARK annotation in gene expression changes upon knockdown of TRRAP, CHD8 or MYC are shown. Experiments are performed in triplicate, averages are shown, compared to control knockdowns.
