# Supplementary material for: A CHD8-TRRAP axis facilitates MYC and E2F target gene regulation in human neural stem cells
Source: iScience. 2025 Feb 12;28(3):111978. doi: 10.1016/j.isci.2025.111978 (PMC11914185; doi:10.1016/j.isci.2025.111978)
Supplement: Document S1. Figures S1 and S2 [file mmc1.pdf]

iScience, Volume *28*

Supplemental information

A CHD8-TRRAP axis facilitates MYC and E2F target gene regulation in human neural stem cells

Lize Meert, Mariana Pelicano de Almeida, Mike R. Dekker, Dick H.W. Dekkers, Karol Nowosad, Danny Huylebroeck, Mirjam van den Hout, Zeliha Ozgür, Wilfred F.J. van IJcken, Jeroen Demmers, Maarten Fornerod, and Raymond A. Poot

Supplemental Figures.

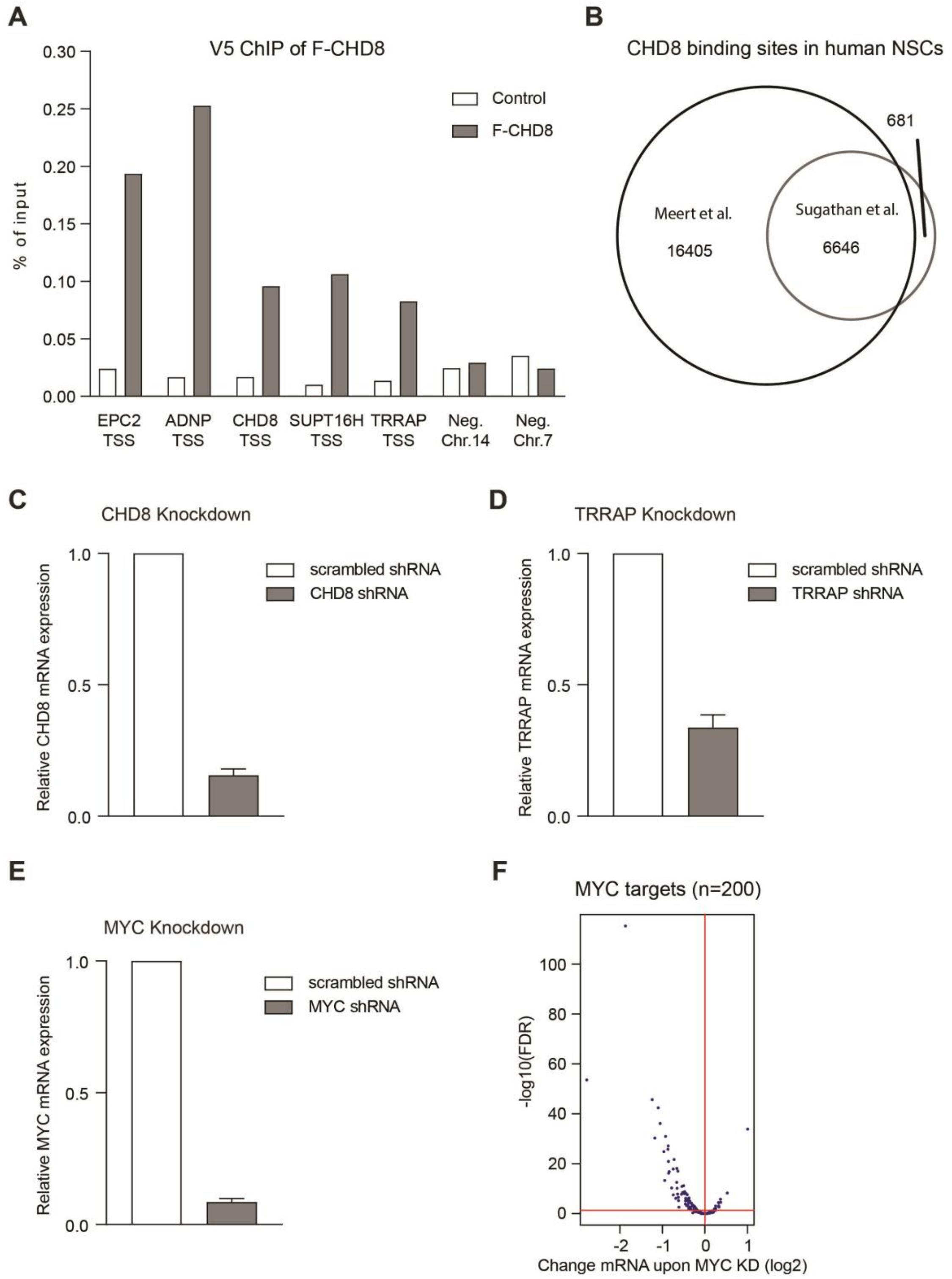

Figure S1. Binding site data and gene expression data, related to Figures 1, 2 and 4.

(A) F-CHD8 ChIP precipitates CHD8 target promoters. Anti-V5 antibody ChIP from F-CHD8 hNSCs and wild type hNSCs. F-CHD8 contains a V5-tag. % of input of V5-ChIP-precipitated

DNA is indicated for the transcription start site (TSS) of the indicated genes from control hNSCs (white) and F-CHD8 hNSCs (grey). Negative control regions on chromosome 14 and 7 are indicated.

(B) Venn diagram of CHD8 significant binding sites in this manuscript (Meert et al.) and CHD8 significant binding sites from Sugathan et al.[18]. Numbers of CHD8 binding sites, overlapping or not, are indicated. Overlap P = 0 by hypergeometric test with the number of possible binding places equal the sum all detected peaks.

(C) Knockdown of CHD8 by RNAi in hNSCs. RT-PCR for relative CHD8 mRNA levels 72 hrs after transfection of plasmids expressing CHD8 shRNA (grey) or scrambled shRNA (white). The error bar depicts the standard deviation and the experiment was performed in triplicates.

(D) Knockdown of TRRAP by RNAi in hNSCs. RT-PCR for relative TRRAP mRNA levels 72 hrs after transfection of plasmids expressing TRRAP shRNA (grey) or scrambled shRNA (white). The error bar depicts the standard deviation and the experiment was performed in triplicates.

(E) Knockdown of MYC by RNAi in hNSCs. RT-PCR for relative MYC mRNA levels 72 hrs after transfection of plasmids expressing MYC shRNA (grey) or scrambled shRNA (white). The error bar depicts the standard deviation and the experiment was performed in triplicates.

(F) Scatterplot showing mRNA changes of GSEA-assigned MYC target genes (n=200, as used in Figures 3A-C and 4A,B) upon knockdown of MYC in hNSCs. Red horizontal line indicates significance threshold (FDR = 0.05).

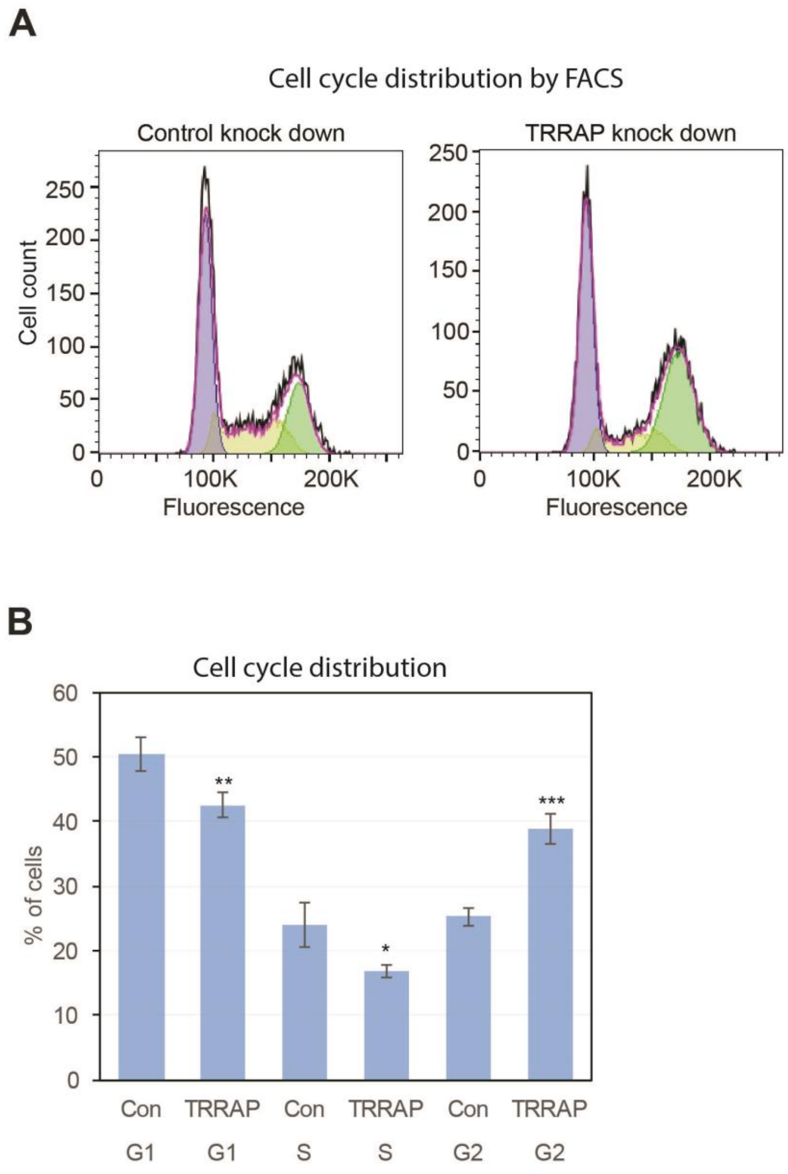

Figure S2. Cell cycle distribution upon TRRAP knock down, related to Figure 4.

(A) Propidium Iodine (PI) FACS of hNSCs with knock down of TRRAP (right panel) or control knock down (left panel). Typical cell count distribution on PI fluorescence is shown. Different colorings indicate (left to right) cells populations in G1 phase, S phase and G2 phase.

(B) Percentages of cells in G1, S and G2 phase. TRRAP indicates hNSCs with knock down TRRAP, Con indicates hNSCs with control knock down. Average of 4 biological replicate experiments and standard deviation is shown. P-values (two-tailed t-test) of differences between the G1, S and G2 fractions of Con and TRRAP are shown; P-value < 0.001 is indicated with ***, P-value < 0.01 is indicated with ** and P-value <0.05 is indicated with *.
